# The Utility of Lighted Ureteral Stents in Laparoscopic Colorectal Resection: A Survey of Canadian Surgeons

**DOI:** 10.4021/gr344w

**Published:** 2011-07-20

**Authors:** Anna M. Borowiec, Richdeep S. Gill, Daniel W. Birch, Shahzeer Karmali

**Affiliations:** a Department of Surgery, University of Alberta, Edmonton, Alberta, Canada; b Center for the Advancement of Minimally Invasive Surgery (CAMIS), Royal Alexandra Hospital, Edmonton, Alberta, Canada; * Co-first author

**Keywords:** Lighted, Ureteric stent, Laparoscopic, Colorectal surgery, Ureteric injury

## Abstract

**Background:**

Establishing the exact location of the ureters is critical in preventing ureteric injury during colorectal surgery. In laparoscopic colorectal resections this identification can be facilitated by the pre-operative insertion of lighted ureteral stents (LUS). LUS may also serve as an invaluable educational aid during the teaching of colorectal surgery. However, the available evidence does not support the routine use of stents as an injury prevention measure. Furthermore, stent insertion carries inherent risks of ureteric injury. The objective of this study was to determine the frequency of use and indications for LUS in laparoscopic colorectal resections among Canadian surgeons.

**Methods:**

A seven-question survey was administered to Canadian surgeons through the monthly Canadian Association of General Surgeons (CAGS) e-news over a period of three months. The questions focused on surgeon demographics, experience with laparoscopic colon resections and the use of stents.

**Results:**

Seventy-five surgeons completed the survey. There was a wide range of experience among the surgeons in terms of years in practice. The majority (84%) reported performing laparoscopic colorectal resections and of those 65% reported performing less than 25 resections a year. Only 26% of surgeons used LUS during laparoscopic resections. Furthermore, 75% of LUS users did not have sub-specialty training, 69% performed less than 25 resections per year and 50% were in practice for less than five years. When used, LUS were inserted for diverticular disease (100%), left colon resection (88%) and low anterior resections (75%).

**Conclusion:**

The majority of surgeons across Canada do not use LUS for laparoscopic colorectal resections. Of those performing laparoscopic colorectal resections, there may be a preference to use LUS for complex cases and by novice operators. This data suggests that proponents of LUS deem that it may have a role in diverticular disease.

## Introduction

Laparoscopic surgery in benign and malignant colorectal disease has been shown to be associated with improved recovery [1-3] and earlier return of bowel function [2-4]. In both open and laparoscopic resection of the colon and rectum, precise identification of the position of the ureters remains an important method to avoid iatrogenic injury. Although relatively uncommon, the incidence of ureteric injuries following colorectal resection is approximately 1-10% [5-8]. Ureteric identification in open colorectal surgery involves palpation and direct visualization of the ureters, whereas in laparoscopic surgery, direct visualization is the only approach. As a result, the use of lighted ureteric stents (LUS) has been suggested [9]. However, the use of ureteric stents as an injury prevention measure remains controversial, with stent insertion carrying inherent risks of hematuria and ureteric injury [5]. Our objective was to determine the frequency of use and factors related to preference for LUS in laparoscopic colorectal resections among Canadian surgeons.

## Methods

An online survey was made available to all 1200 Canadian Surgeons through the monthly Canadian Association of General Surgeons (CAGS) e-news for three months. More specifically the “monthly CAGS e-news” consisted of an email sent out by the association to all members of CAGS and the link for the survey website along with the brief explanation of the survey was embedded in text of the monthly news.

The survey was anonymous and it consisted of the seven following questions focusing on surgeon’s demographics, experience and use of LUS: (1) What is your training? (Do you have subspecialty training?) (2) How many years have you been in practice? (3) Where do you practice? (which province?) (4) Do you perform laparoscopic colon resections? (5) What is the number of laparoscopic left colon/sigmoid resections you do in one year? (6) Do you use LUS for your laparoscopic colon resections? (7) When do you use LUS for laparoscopic colon resections?

For the last question surgeons were asked to choose all applicable answers and answers consisted of type of colorectal resection (right colon, left colon, low anterior resection) and underlying diagnosis (malignancy, diverticular disease, ischemic colitis, inflammatory bowel disease). For all answers multiple choices were given, for which the respondent could choose the best answer. At the conclusion of the three month period, all answers were combined and results expressed as percentages.

## Results

### Surgeon’s experience

**Figure 1:**
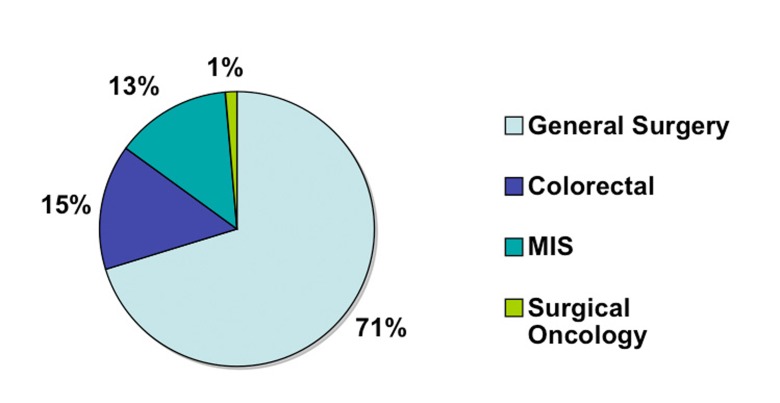
Surgeon training.

**Figure 2:**
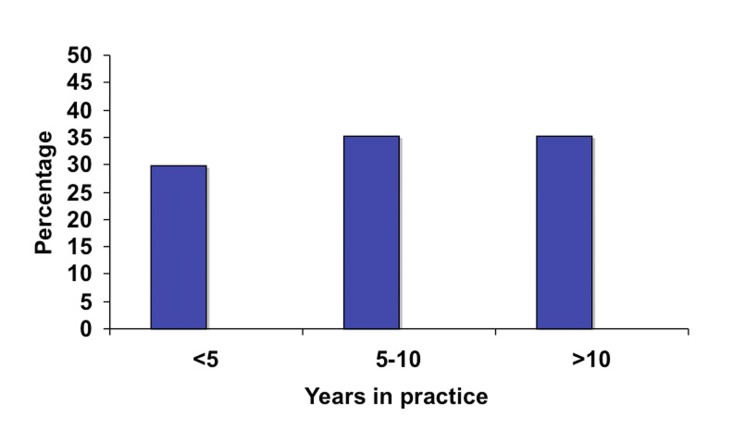
Surgeon experience based on years in practice.

**Figure 3:**
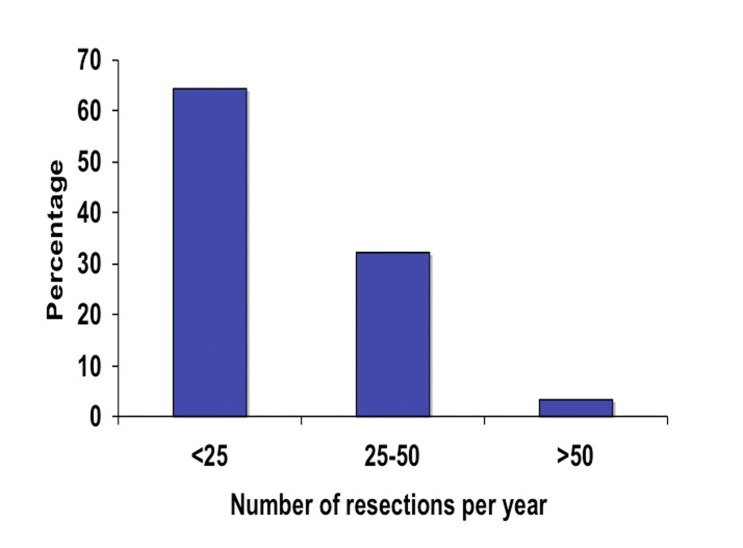
Surgeon experience based on the annual number of laparoscopic colorectal resection.

A total of 75 surgeons completed the on-line survey out of 1200 potential recipients of the questionnaire. This gives us a potential response rate of 6.3%. Of those 75 surgeons, the majority (71%) had no additional subspecialty training aside from general surgery training (Fig. 1). Fifteen percent had additional training in colorectal surgery, 13% had training in minimally invasive surgery (MIS) and 1% in surgical oncology. In terms of years in practice, the surgeons were evenly distributed between three categories of less than five years in practice, five to ten years and more than ten (Fig. 2). Eighty-four percent of responders reported performing laparoscopic colorectal resections and of those the majority (65%) performed less than 25 operations a year and very few surgeons performed more than 50 such operations annually (Fig. 3).

### Use of stents

Only 25% of surgeons said they were using LUS for their laparoscopic resections. When used, LUS were used selectively but most frequently for left colon resections (88%) and low anterior resections (75%). The single most common reason to use LUS was diverticular disease (100% of respondents), followed by malignancy (62%).

**Figure 4:**
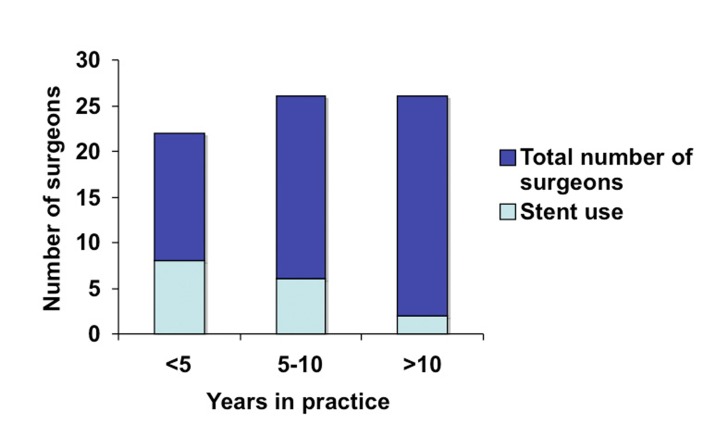
LUS use based on number of years in practice.

**Figure 5:**
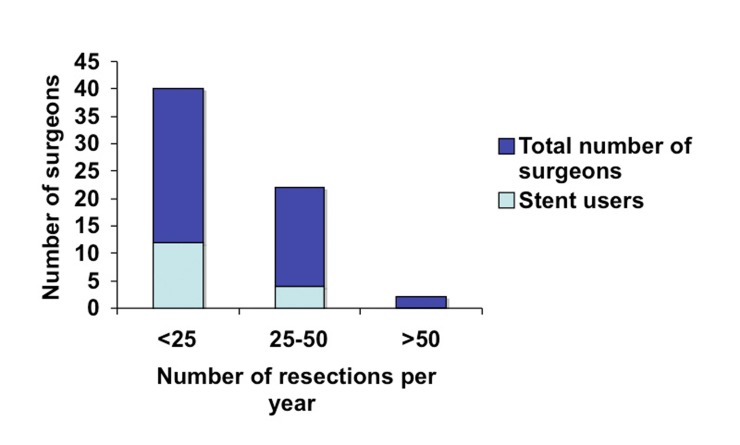
LUS use based on the annual number of laparoscopic colorectal resection.

When LUS use was analyzed according to experience (years in practice and number of laparoscopic colorectal resection per year), we identified greater use among novice surgeons: 40% of surgeons in practice for less than five years used LUS versus 25% of surgeons in practice for five to ten years (Fig. 4). Similarly, a third of surgeons performing less than 25 resections years reported using stents in contrast to a fifth of surgeons who performed 25 to 50 resections annually (Fig. 5).

## Discussion

According to the results of our online survey, the preoperative placement of LUS was utilized by 25% of Canadian surgeons for mostly left colon and low anterior resections, with diverticular disease being the most common indication. Both more novice surgeons and those performing less than 25 surgical resections were identified to more commonly employ LUS.

The pre-operative placement of ureteric stents in colorectal surgery remains a controversial topic among surgeons. An important objective of laparoscopic colorectal surgery is to perform adequate resection with proper identification and preservation of the ureters. Estimated complication rates of ureteric injury are approximately 1 to 10% [5-8]. Ureteric injury often is due to either ligation or transection of one or both ureters. Other complications include ureteral fistulization and stricture. Management of ureteric injuries depends on the site and extent of injury, but time of discovery is essential [10]. With early identification of the ureteric injury, end-to-end anastomosis is possible. However, with delayed identification, more complex reconstructive options need to be explored. Though rare, nephrectomy may not be avoidable in some cases [10, 11]. Some authors suggest that early identification of ureteric injuries may be the primary benefit of ureteric stents [12, 13].

A 12-year randomized trial in gynecologic surgery comparing bilateral prophylactic ureteral catherization to no intervention found that ureteral injury rates were low and similar between both groups (1.2% vs. 1.1%, respectively) [14]. Similarly, Chahin et al reported a ureteral laceration rate of 1.5% in 66 patients following laparoscopic colectomy with LUS [9]. However the single ureteric laceration was successfully managed with reinsertion of the stent. On the other hand, the complications related to stent insertion include hematuria and ureteric injury [5]. The most serious complication is termed reflux anuria, in which severe oliguria or anuria results from manipulation of the ureters resulting in constriction and development of edema [15]. In the same study by Chahin et al, 66 patients that received LUS prior to laparoscopic colectomy, four developed anuria, with two requiring temporary renal support [9]. Sheikh et al reported 3 out of 59 patients developing reflux anuria following prophylactic ureteral catheterization during colorectal surgery [16].

Only 25% of Canadian surgeon respondents are utilizing LUS for laparoscopic colorectal operations. Of these responding surgeons, LUS appears to be used selectively for presumed difficult cases or used during early practice. Bothwell et al concluded from a five-year review that prophylactic ureteric stenting might be reasonable in complicated cases such as complicated diverticulitis [12]. With this study being an Internet survey of Canadian surgeons, we cannot comment on complication rates or effectiveness of LUS in injury prevention during laparoscopic distal colon and rectal surgery. However, the perception from our questionnaire indicates that use of selective LUS in laparoscopic colorectal surgery may be reasonable for more novice operators in complicated colorectal resections (namely, complicated diverticulitis). However, the literature is divisive regarding the safety and efficacy of LUS in laparoscopic colorectal surgery.

There have been no previous studies comparing LUS to conventional ureteral stents. The main benefit of LUS over conventional ureteral stents remains much improved visibility. With laparoscopic surgery relying predominantly on visual cues, LUS may be the superior option. However, there is limited literature comparing LUS and conventional stents in terms of cost and complications, thus we can only speculate on the superiority of LUS for laparoscopic colorectal resection.

There are important limitations of our on-line survey, including a low response rate and response bias. Firstly, most epidemiologic questionnaires aim for a response rate over 30%. Unfortunately, our response rate is only 6.3% from 1200 potential respondents. But it must be noted, that the CAGS membership consists of a diverse group of general surgeons with highly varied practices. It is unlikely that all 1200 potential respondents perform laparoscopic colorectal surgery. Therefore, they would not be included in the final denominator used to determine response rate. Regrettably, it is difficult to determine from the CAGS membership which surgeons perform laparoscopic colorectal surgery. Also, because the questionnaire is anonymous, this question cannot be ascertained after the fact. However, we attempted to maximize responses by repeatedly sending the on-line survey link with CAGS e-news emails. In hindsight, sending the survey via mail may have potentially increased responding Canadians surgeons. Nonetheless, only a small percentage of Canadian surgeon respondents are utilizing LUS for laparoscopic colorectal operations. Thus, a greater response rate may not change the results in terms of general perception, but would strengthen potential conclusions. Secondly, response bias is common in surveys such as ours. Canadian surgeons with an interest in LUS or strong opinions for or against LUS are more likely to respond to the questionnaire. Interestingly, the majority of respondents do not utilize LUS for laparoscopic colorectal operations. Of those using LUS, a majority utilizes them for similar indications. Thus, the perceived indications for LUS in laparoscopic colorectal operation may be more common among Canadian surgeons. However, there is no pre-existing Canadian literature to support this claim.

The use of LUS has not been formally explored for teaching advanced laparoscopic colorectal surgery. It may serve as an important tool in teaching residents and practicing surgeons advanced laparoscopic techniques. The concise localization of the ureters by LUS would aid in dissection by novice operators, especially in the distal colon and rectum. Further research is needed to determine the utility of LUS as education tool.

### Conclusion

Of the responding Canadian surgeons who perform laparoscopic colorectal resections, a minority utilize LUS. However, there may be a preference to use of LUS for distal colon and rectal operation involving diverticulitis. Further research is needed to clarify the role of LUS in laparoscopic colorectal operations.
